# Clinicopathological and molecular characteristics of synchronous gastric adenocarcinoma and gastrointestinal stromal tumors

**DOI:** 10.1038/s41598-017-12622-x

**Published:** 2017-10-10

**Authors:** Jun-Ming Luo, Fa-Long Cao, Chen Meng, Li-Jun Lin, Si-Qing Ma, Shao-Hua Peng, Hong-Ling Gao, Sara Javidiparsijani, Gui-Rong Wang, Meng-Lan Zhang, Jian-Guo Xin, Yi-Chun Wang, Shu-Kun Zhang

**Affiliations:** 1 Department of Pathology, Qinghai Provincial People’s Hospital, Xining, 810007 Qinghai Province China; 2 Department of Dermatology, Qinghai Provincial People’s Hospital, Xining, 810007 Qinghai Province China; 3 SinoMD Gene Technology Co, Ltd, Beijing, 100176 China; 4 Department of Intensive Care Unit, Qinghai Provincial People’s Hospital, Xining, 810007 Qinghai Province China; 5 Department of Pathology, Medical School of Hunan Normal University, Changsha, 410007 Hunan, Province China; 6 Department of Surgery, SUNY Upstate Medical University, Syracuse, NY13210 USA; 7 Department of Anesthesiology, Hunan Provincial Tumor Hospital, the Affiliated Tumor Hospital of Xiangya School of Medicine, Central South University, Changsha, 410013 Hunan Province China; 8 Department of Pathology, Weihai Municipal Hospital, Weihai, 264200 Shandong Province China

## Abstract

Synchronous gastric tumors that consist of both gastrointestinal stromal tumor (GIST) and adenocarcinoma are rare. We studied the clinicopathological and molecular characteristics of six cases containing both gastric adenocarcinoma and GIST. By means of immunohistochemical analysis, all GIST cells expressed CD117, CD34 and Dog1 in all six synchronous gastric adenocarcinomas with GIST, and in GIST alone. Sequencing analysis demonstrated that exon 11 *c-kit* mutations were present in two of six synchronous tumors and four of five GISTs. One of the two exon 11 *c-kit* mutations in synchronous adenocarcinomas with GISTs was an uncommon mutation of CTT > CCA at amino acid 576, and the other was a GTT deletion at amino acid 560. The mutation was a homozygous A > G mutation in exon 12 (amino acid 567) of *PDGFR-α*. We concluded that the exon 11 mutations were the most important in both cases of synchronous gastric adenocarcinoma with GIST and GIST alone. The mutation rate was higher in GIST alone than in synchronous adenocarcinoma with GIST.

## Introduction

Gastrointestinal stromal tumor (GIST) is the most common mesenchymal tumor of the gastrointestinal tract, and because of its CD117 positive phenotype, it probably originates from Cajal cells^1–3^. GIST usually occurs in older individuals with a median age of 60–65 years with no difference between men and women. GIST is common in the stomach^1–3^. Benign cases of GIST are more frequent than malignant cases. However, the small GIST can be found occasionally^4,5^.

GIST can occur in any area of the stomach but predominantly at the outside of the gastric lumen or the lining of the stomach, as well as being attached to the lining of the stomach^4,5^. The size of GISTs varies from case to case. Tumor cells have a wide morphological spectrum, although most are fusiform; 20–25% of GIST cases have both fusiform and epithelial tissue types, and some cases show mixed histological appearance. Most GISTs are positive for *c-kit* (CD117) with strong expression of CD34 and Dog1 and weak expression of smooth muscle actin. Only a few cases of GIST express actin, CK18 or S-100^4–6^.

The gene homology of *PDGFR-α* and c-*kit* is high. The c-*kit* is located on chromosome 4q12-13, as a proto-oncogene and its product is type III tyrosine kinase. Expression of *c-kit* (a proto-oncogene receptor) can combine with somatic cell factor and stimulate the phosphorylated tyrosine residue that regulates cell growth and tumor proliferation, malignant evolution, and apoptosis.

*PDGFR-α* gene encodes a single transmembrane glycoprotein that is involved in mitosis and other signal transmission into the nucleus, thus causing cell division and proliferation. Mutations of *PDGFR-α* can lead to malignancy. The *c-kit* or *PDGFR-α* mutations cause functional changes and are thought to be major molecular mechanisms of GIST.

About 65–90% of GISTs have either *c-kit* or *PDGFR-α* mutation. Exon 11 mutation of *c-kit* is more common than mutations in exons 9, 12, 13, 14, 17 and 18. Exon 11 is a highly conserved region located in the juxtamembrane domain (amino acids 543–580) between the transmembrane domain (amino acids 521–543) and kinase domain (amino acids 581–936).

There is normally a *PDGFR-α* mutation in GISTs with wild-type *c-kit*. The mutation rate of *PDGFR-α* is lower than that of *c-kit*, and only a few GISTs have both *c-kit* and *PDGFR-α* mutation. *PDGFR-α* mutation usually occurs in exon 18 and causes an amino acid change (D842V), but is also observed in deletion of exon 12 and the mutation of exon 14.

Gastric cancer accounts for ~7.8% of all types of cancer. More than 700,000 individuals die from stomach cancer each year, and it is ranked as the second most frequent cause of cancer mortality worldwide. About 974,000 new cases of gastric cancer are diagnosed annually, which makes it the fourth most common malignant tumor worldwide. Gastric cancer occurs mainly in elderly people and rarely in those under the age of 30 years. Gastric cancer is associated with multiple factors including smoking, diet, bile reflux, and *Helicobacter pylori* infection. The WHO classifies gastric cancer histologically as tubular, papillary, myxoid, low adhesion carcinoma (including signet ring cell carcinoma), and mixed carcinoma.

Although mixed adenocarcinoma with other tumors in the stomach is rare several cases have been reported previously, in which synchronous tumors of the stomach consist of adenocarcinoma mixed with gastric lymphoma^7–10^, as well as with a carcinoid tumor^9,11,12^. However, gastric synchronous tumor consisting of adenocarcinoma with GIST is rare. Ruka *et al*.^13^ found that about 10% of their GIST patients were an associated non-GIST neoplasm, like carcinoma. Furthermore, Maiorana *et al*.^14^ reported that among 52 patients with gastric GIST, six (11.5%) were an associated, second gastric tumor (five adenocarcinomas and one carcinoid tumor).

To date, most of the publications about GISTs have been single case reports. Here, we present six patients with synchronous gastric adenocarcinoma with GIST. The aim of this study was to evaluate the clinicopathological and molecular oncogenesis characteristics of GIST occurring concomitantly with gastric adenocarcinoma.

## Results

### Patients

We included five male and one female patients with a median age at presentation of 61.3 years (range, 47–71 years) in this study. The common clinical manifestations were abdominal discomfort (n = 3), backache (n = 2) and difficulty eating (n = 1). Some patients had more than one of these symptoms. The median duration of disease was 1.5 months (range, 0.3–6 months). All six patients underwent preoperative gastroscopy, which revealed ulcerative lesions in four, an elevated lesion in the antrum and an erosive lesion in the cardia. One patient had a soft tissue lesion with a diameter of 1.5 cm in the lesser curvature, which was considered to be a GIST. All lesions were diagnosed as adenocarcinomas on biopsy examination. Computed tomography (CT) and chest images were available in all the six patients. Total (n = 2) and subtotal (n = 3) gastrectomy, and partial gastrectomy with partial oesophagectomy (n = 1) were performed.

### Clinicopathology

The locations of adenocarcinoma in the stomach are shown in Table 1. The mean size of primary adenocarcinoma was 4.4 ± 2.0 cm (range, 2.0–7.5 cm). There were no distant metastases in any of the patients at the time of diagnosis. Three of them were low adhesion carcinoma, and the other three were moderately differentiated tubular adenocarcinomas.

**Table 1:** Clinical characteristics and pathological features of gastric epithelial tumors.

Case No.	Sex/age, yr	Preoperative diagnosis	Surgery	Site	Size, cm	Gross appearance	Histology	Stage (pTNM)
1	M/47	Adenocarcinoma	PG	Antrum	5.5	Ulcer	Moderately differentiated adenocarcinoma	T4b N3a M0
2	F/60	Adenocarcinoma	PG	Corpus	3	Ulcer	Low adhesion cancer	T3 N3a M0
3	M/64	Adenocarcinoma	PG	Antrum	2	Mass	Moderately differentiated adenocarcinoma	T3 N0 M0
4	M/59	Adenocarcinoma	TG	Fundus	5.5	Ulcer	Low adhesion cancer	T2 N0 M0
5	M/67	Adenocarcinoma	PG+PO	Cardia	3	Ulcer	Moderately differentiated adenocarcinoma	T2 N0 M0
6	M/71	Adenocarcinoma	TG	Corpus	7.5	Erosion	Low adhesion cancer	T4a N3b M0

PG: partial gastrectomy; PO: partial oesophagectomy; TG: total gastrectomy.

**Figure 1:**
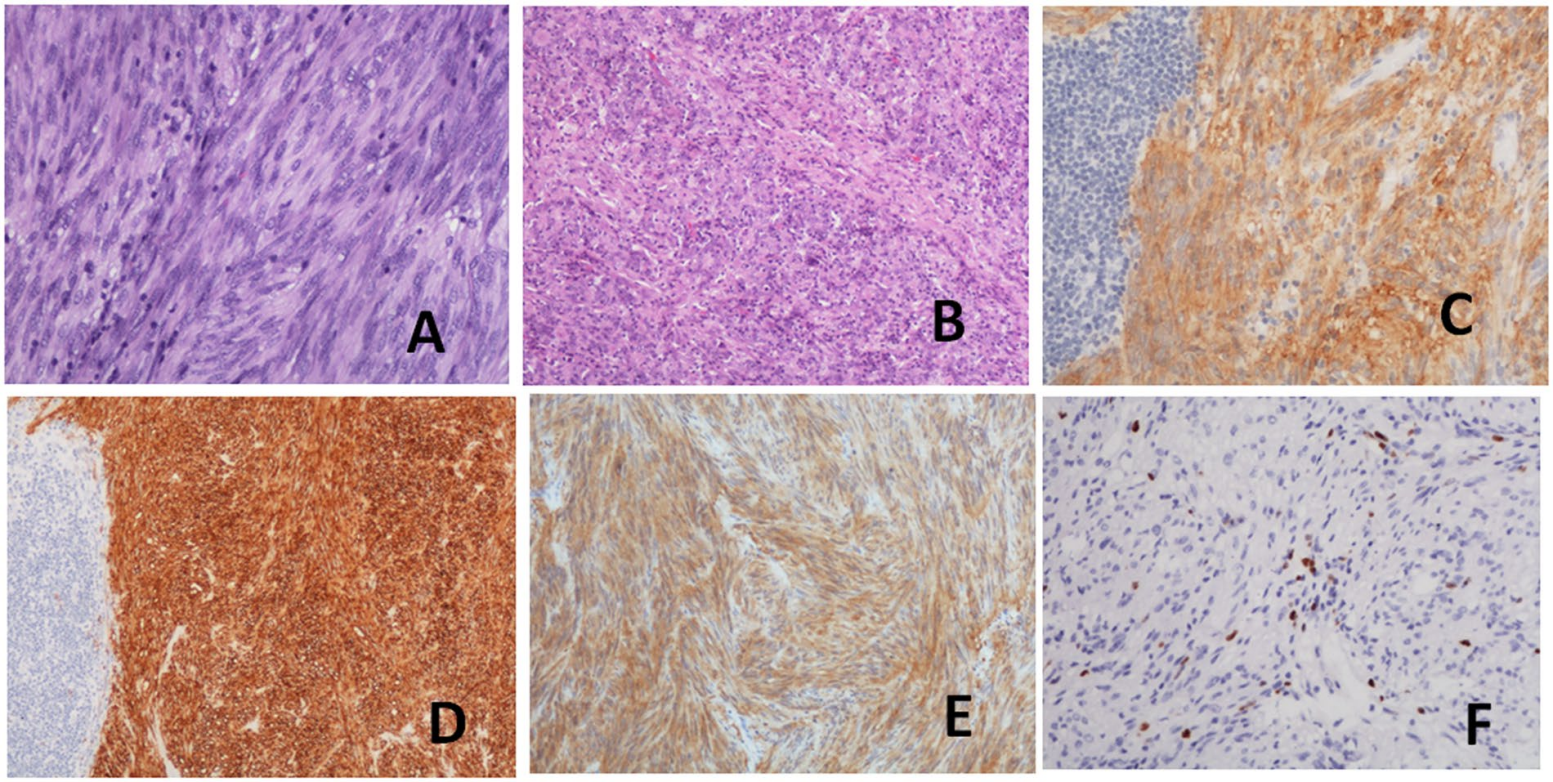
Morphological and immunohistochemical characteristics of synchronous. Adenocarcinoma and GISTs were composed of spindle (**A**), and epithelioid (**B**) cells in H&E staining, and showed strong expression c-Kit CD117 (**C**), CD34 (**D**), and Dog1 (**E**), and low expression of Ki-67 (**F**). H&E: haematoxylin and eosin.

Five of the GISTs were an incidental finding during surgery or gross pathological examination after surgery. Detailed clinicopathological data are shown in Tables 1 and 2. The mean GIST size was 1.0 ± 0.4 cm (range, 0.5-1.5 cm). Five of six GISTs were located in the submucosal layer and only one in the intramural layer. All GISTs were of the spindle cell type and were strongly and diffusely positive for Dog-1, CD34 and CD117 markers (Fig. 1). Four of six GISTs were also positive for vimentin (66.7%) and four of them for S-100. Additionally, Ki-67 index for the GISTs was <5% in all cases. And all cases had a low risk of recurrence.

**Table 2:** Pathologic features and DNA mutation of gastric stromal tumors.

Case No.	Site	Size, cm	Gross appearance	Histology	Codon	DNA mutation	Amino acid
1	Corpus	0.5	Submucosal nodule	Spindle cell, very low risk of recurrence	c-kit 11	CTT > CCA	576
2	Corpus	0.8	Submucosal nodule	Spindle cell, very low risk of recurrence	Wild type		
3	Corpus	1.5	Intramural mass	Spindle cell, very low risk of recurrence	Wild type		
4	Corpus	1.0	Submucosal nodule	Spindle cell, very low risk of recurrence	c-kit 11	deleted GTT	560
5	Fundus	0.8	Submucosal mass	Spindle cell, very low risk of recurrence	Wild type		
6	Corpus	1.5	Submucosal nodule	Spindle cell, very low risk of recurrence	Wild type

### Oncogenes and their mutations

**Figure 2:**
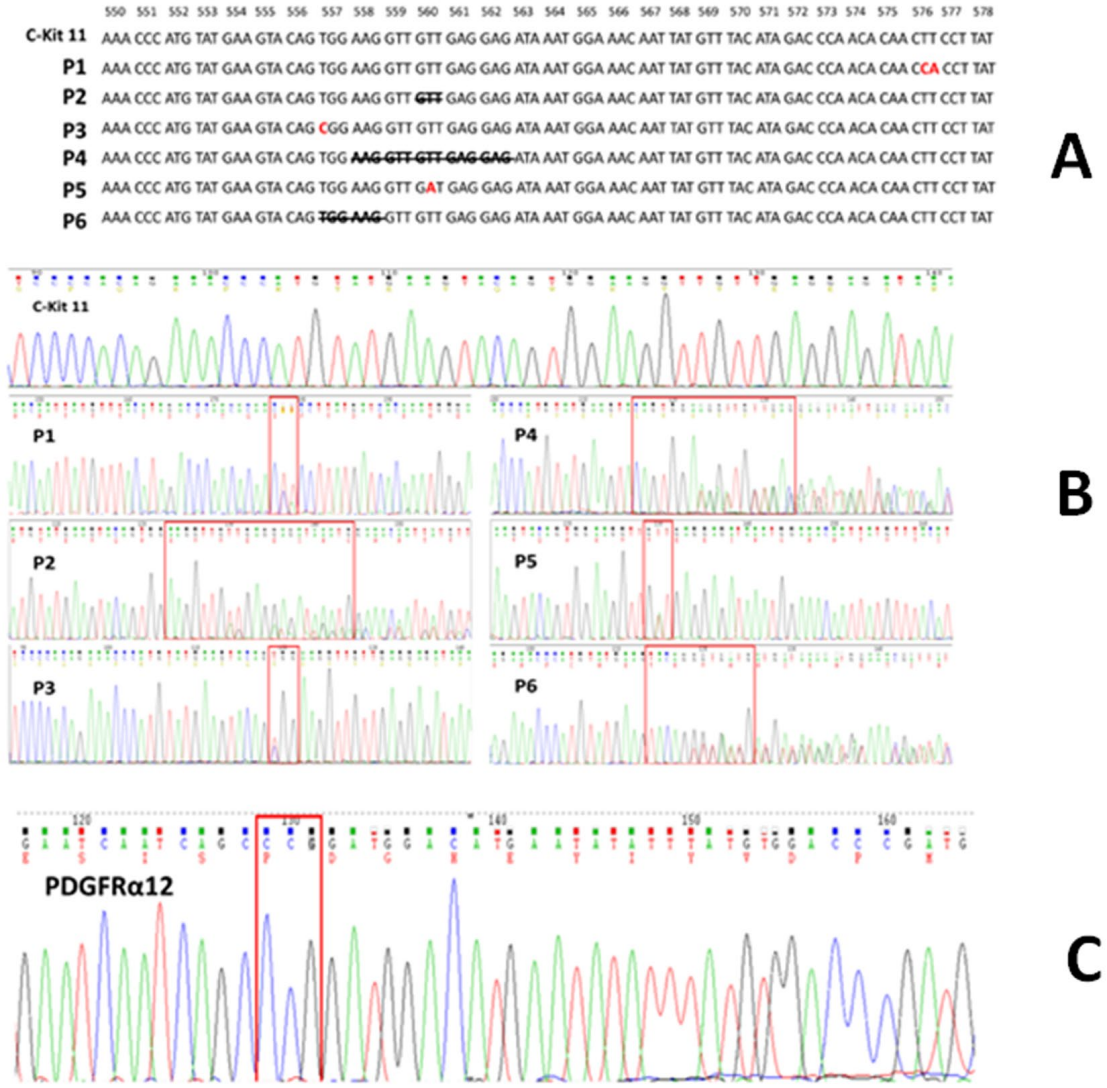
Oncogenic characteristics of synchronous adenocarcinoma and GIST. Direct sequencing of *c-kit* exon 11 (**A**,**B**) and *PDGFR-α* (**C**). In the six synchronous cases, we found two mutations in exon 11 of *c-kit*: one uncommon mutation of CTT > CCA leading to mutation of amino acid 576, and GTT deletion leading to deletion of amino acid 560 [Patient 1 (**A**) and Patient 2 (**B**)]. In the five GIST alone cases, four had *c-kit* exon 11 mutations: W > R mutation at amino acid 557 (**B**; Patient 3); deletion mutation of amino acids 558–562 (**B**,**C**; Patient 4); V > D mutation resulting in deletion of amino acid 560 (**A**,**B**; Patient 5); and deletion mutation of amino acids 557–558 (**A**,**B**; Patient 6). A homozygous A > G mutation was also found in exon 12 of amino acid 567 of *PDGFR-α* (**C**).

Paraffin-embedded tissue specimens for all six synchronous adenocarcinomas with GISTs and GISTs alone were screened for mutations in *c-kit* and *PDGFR-α*. Two of six (33.3%) synchronous tumors had *c-kit* mutations, and four of five (80%) GISTs had *c-kit* exon 11 mutations (Fig. 2A,B). There was a homozygous A > G mutation in exon 12 of amino acid 567 in all GISTs with adenocarcinoma and GISTs alone (Fig. 2C). There were no mutations in other *c-kit* exons (9, 12, 13, 14, 17 and 18) or exons 14 and 18 of *PDGFR-α*.

Among the two synchronous tumors with exon 11 *c-kit* mutations, one had an uncommon mutation of CTT > CCA at amino acid 576, and the other had a GTT deletion that resulted in deletion of amino acid 560 (Fig. 2B,C). In the five cases of GIST alone, four had *c-kit* exon 11 mutations: W > R mutation at amino acid 557, deletion mutation of amino acids 558–562, deletion mutation of amino acids 557–558, and V > D mutation resulting in deletion of amino acid 560 (Fig. 2B,C). Only one case had wild-type *c-kit* in exon 11.

## Discussion

GIST was first mentioned in 1983 by Mazur *et al*.^15^. GIST is a common mesenchymal tumor that originates in the digestive tract^16^. The stomach is the most common site of GIST, followed by small intestine, colorectum and oesophagus. The clinical manifestations of these tumors depend on their locations and sizes^17,18^.

The six cases in the present study were patients undergoing stomach cancer surgery, with clinical symptoms caused by gastric cancer, including abdominal discomfort, back pain and difficulty swallowing. Most cases of GIST were accidentally discovered by imaging, surgeons during operation, or pathologists after operation. Only one of the six cases was found by preoperative imaging. However, preoperative imaging can find other coexistent lesions in the stomach. Surgeons need to check carefully for other lesions in the stomach and remove them for frozen pathological examination. In addition, pathologists need to assess carefully the lining of the stomach to rule out the possibility of small GISTs.

Symbiosis of gastric cancer with other tumors is rare, with a reported incidence of 4.5–35%^19–26^; gastric adenocarcinoma is the most common (47%), followed by lymphoma (7%). In a retrospective study of 60 cases with 22 GISTs and other tumors, there were only two cases of GIST with gastric cancer.

The occurrence of single cases of adenocarcinoma mixed with gastric lymphoma, carcinoid tumor, leiomyosarcoma^14,27–30^ or rhabdomyosarcoma^27,28^, as well as sarcomatous stromal components, has been reported^31^. So far, only a few reports of gastric collision tumors with synchronous adenocarcinoma and leiomyoma have been documented^32,33^. In the literature, there are only a few cases of concurrent presentation of gastric adenocarcinoma and GISTs^14,18,19,30,34–39^.

GISTs are usually composed of spindle-shaped or epithelioid cells or a mixture of both. The immunohistochemistry of GISTs is positive for *c-kit* expression (CD117) and often for CD34 and Dog-1, and occasionally the cells are positive for smooth muscle actin, desmin and S-100 expression. In the present study, all GISTs were strongly and diffusely positive for Dog-1, CD34 and CD117. Four of them were also positive for vimentin and four for S-100. The two most important prognostic factors are tumor size and mitotic index^40^. According to this classification, all six patients in this study had low or very low risk of locally advanced tumor or metastasis. The biological behaviour might have been concealed by gastric cancer. However, definitive evidence for this need to be investigated in the future and careful follow-up is mandatory.

Approximately 85% of GISTs are associated with an abnormal *c-kit* pathway. In this study, we only found two *c-kit* mutations among the six cases of synchronous adenocarcinoma with GIST, which was lower than in the GIST alone group (four of five patients). CD117 is a transmembrane receptor for stem cell factor, and consists of a long extracellular domain, transmembrane segment, and intracellular part. The common mutations in GISTs are located in exons 9 and 11, and rarely in exons 12, 13, 17 and 18 of *c-kit*. The *c-kit* also codes for tyrosine kinase functions that are important in therapy for GISTs. Most *c-kit* wild-type GIST patients have a mutation in another gene, such as *PDGFR-α*, which is also related to tyrosine kinase. Mutations in *c-kit* and *PDGFRα* are mutually exclusive. Previous studies have shown that few GISTs appear to be associated with neither *c-kit* nor *PDGFR-α* abnormality. Only 10–15% of GISTs carry wild-type sequences in all hot spots of *c-kit* and *PDGFR-α*. These tumours are currently defined by having no mutations in exons 9, 11, 12, 13, 17 and 18 of *c-kit* and exons 12, 14 and 18 of *PDGFRA*. In the present study, there were four *c-kit* exon 11 mutations in the five patients with GISTs alone, which is consistent with other studies, but there were only such mutations in two of six cases of synchronous adenocarcinoma with GIST. It is possible that, in our collision tumors, *c-kit* mutations were influenced by the presence of adenocarcinoma. We will need to collect more cases and investigate the relationship between the molecular structure of *c-kit* and *PDGFR-α* abnormality and oncogenesis.

The relationship between gastric epithelial and stromal tumors is not clear yet. Whether such an occurrence is a simple incidental association or whether the two lesions are connected by a causal relationship remain unresolved. It was believed that this simultaneous presentation was incidental. However, previous studies demonstrated that potential unknown carcinogens may stimulate the simultaneous proliferation and oncogenesis in both epithelial and stromal cells^7,9,17,31,32,34,35^. Although genetic mutations are most important in the pathogenesis of gastric collision tumors there is no direct evidence to support a common genetic mutation underlying gastric adenocarcinoma and GISTs at this stage^37,38,41^. An experimental model also suggested that the carcinogenic agents might interact with two adjacent tissues, which may cause the simultaneous development of tumors of different histological types^7,31,39,42^. For instance, Cohen *et al*.^43^ found that exposure to both acetylsalicylic acid and nitrosoguanidine could induce development of both gastric cancer and leiomyosarcoma. Therefore, they hypothesized that a single carcinogenic agent may interact with two neighboring tissues, inducing different histological cell types in the same organ to develop tumors^43^. For example, *H. pylori* could be a possible carcinogenic candidate that is related to the pathogenesis of gastric carcinoma and mucosa-associated lymphoid tumour^44–46^ or GISTs^17^. It was also hypothesized that the stomach is induced by the unknown carcinogen due to simultaneous proliferation of epithelial and stromal cells^32^.

There were some limitations to our study due to a small number of cases reported. But the results from this study indicated significant difference. Furthermore, we were not able to answer the question of whether a causal relationship exists between gastric adenocarcinoma and GISTs, because we did not study the mutated c-kit protein. Recombinant mutated c-kit protein and biological functions may provide some important information in this regard. However, our results provide an interesting observation that there is a possible association between gastric adenocarcinoma and GISTs. This will be necessary to further validate and investigate using various approaches^47–54^.

In summary, because gastric adenocarcinoma is the major lesion in the majority of cases of adenocarcinoma synchronous with GISTs, the GIST lesion is usually discovered incidentally during imaging or surgery. We report six cases of synchronous gastric adenocarcinoma with GIST, and only one was diagnosed preoperatively by CT. All six GISTs were positive for CD117, Dog1 and CD34. It is difficult to determine the association between GISTs and adenocarcinoma at this stage, although this association is most likely coincidental. Surgical excision is the mainstay of treatment and further research is required to explain this simultaneous tumor development. The *c-kit* mutations are less common in cases of synchronous adenocarcinoma with GIST than in GIST alone.

## Methods

### Patients

We analysed histologically tissues from six consecutive patients (five men and one woman) with CD117-immunopositive synchronous adenocarcinoma with GIST who underwent surgery with curative intent for a primary, resectable tumor between March 2011 and July 2014. Their clinicopathological data were reviewed by two pathologists (S.Z. and J.L.). A review of all available medical and histopathological records was collected from Qinghai Provincial People’s Hospital (Table 1). Patients with postoperative pathological diagnosis of primary gastric adenocarcinoma with GISTs were selected. We excluded patients who had synchronous gastric GISTs and extragastric neoplasms; patients whose initial surgery was performed in other hospitals; and patients with insufficient data. None of the patients had a family history of gastrointestinal carcinoma or GISTs.

### Ethics approval

This study was approved by the Bioethics Committee at Qinghai Provincial People’s Hospital, Xining, China, and informed consent was not required for the retrospective analysis of clinical data.

### Clinicopathology

The gastric adenocarcinomas of each patient were staged according to the TNM system devised by the International Union Against Cancer (Table 1). The risk of aggressive GISTs was assessed using criteria derived from the 2002 Fletcher Classification (Table 1).

Representative haematoxylin and eosin-stained slides of archival tumour specimens were prepared from buffered formalin-fixed, paraffin-embedded tissue blocks. All the antibodies were purchased from MXB Biotechnologies (Fuzhou, China). To confirm the diagnosis of GISTs, immunohistochemical panels were obtained with the following markers: CD117 (Monoclonal 2E4, Cat # MAB-0590, ready-to-use), CD34 (Monoclonal QBEnd/10, Cat # Kit-0004, ready-to-use) and Dog1 (Monoclonal SP31, Cat # Kit-0035, ready-to-use). The immunohistochemical studies were carried out using formalin-fixed, paraffin embedded blocks and primary antibodies to the above biomarkers, on a standard, automated, streptavidin–biotin peroxidase-detection system (EnVision Autostainer Visualization System; Dako Cytomation, Glostrup, Denmark) with a microwave antigen-retrieval step. Parallel positive controls were used for each antibody. A rabbit or mouse, universal, negative-control monoclonal antibody was applied for each specific antibody.

### Oncogenes

Five to 10 sections of 5 µm were placed in an Eppendorf tube. Genomic DNA from GIST or adenocarcinoma areas of synchronous tumours and GISTs alone were extracted using a commercial kit (Tiangen Biotech Co. Ltd., Beijing, China). Gene sequencing for *c-kit* exons 9, 11, 12, 13, 14, 17 and 18 and *PDGFR-α* exons 12, 14 and 18 was performed by Sino-MD Gene Technology Co. Ltd. (Beijing, China). Purified polymerase chain reaction (PCR) amplification products were purified and sequenced. Gene mutations were determined by comparing wild-type sequences. Samples that contained mutations were further examined for the presence of the wild-type *c-kit* gene by subcloning the purified PCR products using a TA cloning vector system (Stratagene, La Jolla, CA, USA). Six independent subclones from each PCR were sequenced by 3500 Dx Series Genetic Analyzer (Applied Biosystem B.V. Singapore).

### Statistical analysis

Statistical analyses were performed using SPSS version 11.0 (Chicago, IL, USA). Descriptive data are presented as the mean ± SD. It was considered significant difference When *P* < 0.05.
